# Active video games for improving health-related physical fitness in older adults: a systematic review and meta-analysis

**DOI:** 10.3389/fpubh.2024.1345244

**Published:** 2024-04-17

**Authors:** Nuannuan Deng, Kim Geok Soh, Borhannudin Bin Abdullah, Hermione Tan, Dandan Huang

**Affiliations:** ^1^Department of Sports Studies, Faculty of Educational Studies, Universiti Putra Malaysia, Serdang, Selangor, Malaysia; ^2^School of Computer Information Sciences, University of the Cumberlands, Williamsburg, KY, United States; ^3^College of Physical Education, Chongqing University, Chongqing, China

**Keywords:** aging, active video games, health game, older adults, physical fitness

## Abstract

**Background:**

The global population is experiencing a rapid rise in the quantity and percentage of older people. In an effort to enhance physical activity among older adults, active video games (AVGs) are being suggested as a compelling alternative and are currently under scrutiny to evaluate their efficacy in promoting the health of older people.

**Objective:**

This review aims to synthesize current studies and formulate conclusions regarding the impact of AVGs on the health-related physical fitness of older adults.

**Methods:**

Seven databases (PubMed, Web of Science, SCOPUS, SPORTDiscus, EMBASE, MEDLINE, and CINAHL) were searched from inception to January 21, 2024. Eligible studies included randomized controlled trials examining the effect of AVGs compared to control conditions on health-related physical fitness outcomes in older adults. The methodological quality of the included trials was assessed using the PEDro scale, and the certainty of evidence was evaluated using the GRADE approach. A random-effects model was used to calculate effect sizes (ES; Hedge’s g) between experimental and control groups.

**Results:**

The analysis included 24 trials with a total of 1428 older adults (all ≥ 60 years old). Compared to controls, AVGs produced significant increases in muscular strength (moderate ES = 0.64–0.68, *p* < 0.05) and cardiorespiratory fitness (moderate ES = 0.79, *p* < 0.001). However, no significant effects were found for body composition (trivial ES = 0.12–0.14; *p* > 0.05) and flexibility (trivial ES = 0.08; *p* = 0.677). The beneficial effects of AVGs were greater after a duration of ≥ 12 vs. < 12 weeks (cardiorespiratory fitness; ES = 1.04 vs. 0.29, *p* = 0.028) and following ≥ 60 minutes vs. < 60 minutes of session duration (muscular strength; ES = 1.20–1.24 vs. 0.27–0.42, *p* < 0.05).

**Conclusion:**

AVGs appear to be an effective tool for enhancing muscular strength and cardiorespiratory fitness in older adults, although their impact on improving body composition and flexibility seems limited. Optimal improvement in cardiorespiratory fitness is associated with a longer duration of AVGs (≥ 12 weeks). Moreover, a session duration of ≥ 60 minutes may provide greater benefits for the muscular strength of older adults.

**Systematic review registration:**

https://www.crd.york.ac.uk/prospero/display_record.php?RecordID=482568, identifier CRD42023482568.

## Introduction

The older population (aged 60 and above) is experiencing the most rapid global growth and is projected to comprise one-fourth of the adult population by 2050 (1, 2). The aging process has been found to diminish the performance of health-related physical fitness (e.g., muscular strength, muscle mass, cardiorespiratory fitness) (3, 4). Such declines are associated with an increased risk of cardiovascular and metabolic diseases, contributing to an overall heightened risk of mortality throughout one’s lifespan (5–7). The components of health-related physical fitness include body composition, muscular strength, muscular endurance, cardiorespiratory fitness, and flexibility (8). Previous research consistently demonstrates a connection between health-related fitness factors and the risk of disease, as well as the quality of life in older adults. For example, lower cardiorespiratory fitness levels are associated with escalated healthcare expenses, decreased life expectancy, and poorer clinical results among older people (9). Muscular weakness is pivotal in developing frailty and functional limitations associated with aging, contributing to various disease processes (10). Therefore, implementing effective intervention strategies to enhance health-related physical fitness could prove highly advantageous for older adults (11).

Engaging in physical exercise has proven effective in reducing frailty and enhancing physical fitness among older adults (12–15). However, traditional forms of physical exercise lack enjoyment, potentially resulting in low adherence rates among older adults for sustained, long-term engagement (16, 17). Active video games (AVGs) have become popular among older people in recent years. These games represent a new generation designed to boost physical activity and reduce sedentary behavior in older individuals (18–20). Indeed, AVGs require players to respond to in-game situations using body movements and gestures, involving full-body motions for interaction in gameplay (21). Integrated with specialized hardware and software, AVGs have the potential to become cost-effective and widely accessible exercise platforms, enhancing health outcomes across diverse populations (22). Older individuals identify enjoyment, social interaction, and perceived health benefits as motivators for staying active (23). A noteworthy aspect of AVGs includes providing immediate auditory and visual feedback on the player’s performance and introducing an enjoyable and motivational element (24). Playing AVGs increases energy expenditure, and the energy expended by older people during AVG sessions is comparable to that of light-to-moderate-intensity activity (21). This can contribute to helping older individuals achieve positive health outcomes (25, 26).

A great number of AVG-related systematic reviews have recently been published. However, the majority of reviews on the topic have concentrated on preventing obesity and promoting physical activity in children and adolescents (27–32). Nevertheless, there have been few meta-analyses that specifically examine the effects of AVGs on older people. Yen et al. (33) and Drazich et al. (34) conducted meta-analyses to assess the impact of AVGs on mental health outcomes. Suleiman-Martos et al. (35) investigated the effect of AVGs on physical function outcomes (e.g., gait speed, strength, and mobility) in older adults living independently in their communities. Pacheco et al. (36) and Taylor et al. (37) performed syntheses on the impact of AVGs on older adults’ mobility and balance. Alhasan and colleagues (38) conducted a meta-analysis evaluating whether AVGs were effective in managing falls and improving postural control in older individuals. Fang et al. (39) gathered randomized controlled studies to examine the effects of AVGs on the balance of healthy older adults. Indeed, these reviews either overlooked additional health indicators (e.g., body composition, cardiorespiratory fitness, and flexibility) or only addressed a small subset of outcome variables in their meta-analysis. Moreover, the absence of moderator analyses (e.g., effects of AVGs according to parameters such as length, frequency, or session duration) in these reviews has limited our understanding of the variables that may affect the main result. Furthermore, others have examined the impact of AVGs on the physical performance of older individuals, but no meta-analysis has been undertaken (40–42). In addition, considering the popularity of AVGs among older people, the effects of AVGs on overall health-related physical fitness have not been systematically reviewed, despite many individual investigations in this field. Therefore, the primary objective of this review is to comprehensively analyze and evaluate existing research on the impact of AVGs on health-related physical fitness in older adults, drawing conclusions through a fair comparison of the included trials. Additionally, our interest also extended to examining potential moderating factors associated with AVG variables, such as length, frequency, and session duration.

## Methods

### Materials and methods

The present review is reported following the updated PRISMA statement (43), and the review protocol has been registered in the PROSPERO (identifier CRD42023482568).

### Search strategy

We systematically conducted a search using specific keywords across seven databases (PubMed, Web of Science, SCOPUS, SPORTDiscus, EMBASE, MEDLINE, and CINAHL) to identify peer-reviewed journal articles in English from the inception of the databases until November 13, 2023. Additionally, we performed an updated search on January 21, 2024, to incorporate more recent works that may not have been included in the synthesis papers. Boolean search strategies incorporated the following search terms: (“older adult” OR senior OR elder OR older adults OR “older person” OR “older people” OR gerontological OR geriatric) AND (“virtual reality” OR exergam* OR “video game” OR Wii OR Kinect OR “X-box” OR Nintendo OR PlayStation OR “dance dance revolution” OR “balance board”) AND (“physical fitness” OR “body composition” OR “body mass” OR BMI OR “body fat” OR “cardiorespiratory fitness” OR “cardiorespiratory endurance” OR “muscular fitness” OR “musculoskeletal fitness” OR “muscle strength” OR “muscular endurance” OR “flexibility”). Moreover, a thorough manual search was conducted on both Google Scholar and the reference lists of all selected papers to ensure that no relevant publications were missed. The search strings used for each database can be found in Supplementary Appendix A.

### Eligibility criteria

The studies were chosen based on the following criteria: (a) the study focused on healthy older adults (≥ 60 years); (b) the training group participated in AVG, described as a technology-driven, screen-based activity that required players to respond to in-game scenarios using gestures and body movements; (c) the study included a control group (e.g., no intervention, usual activity, or education); (d) the study involved one or more health-related physical fitness performance outcomes; and (e) the study was a randomized controlled trial.

The studies were excluded if (a) the older adults who participated in the study either belonged to a clinical population (e.g., Parkinson’s disease, Alzheimer’s disease, stroke, or epilepsy) or were mixed with participants under the age of 60; (b) the AVG intervention was combined with other aerobic training (e.g., Tai-Chi, resistance training), in order to avoid contamination of the AVG effect from other interventions; (c) the study lacked a control group; (d) the study did not offer enough data (i.e., baseline and/or follow-up data) to calculate the effect size (ES).

In the study selection process, an initial review of all relevant titles was followed by thorough evaluations of the abstracts and full texts. Two authors (ND and DH) independently evaluated the collected studies’ titles, abstracts, and full texts. Any disagreements on the inclusion or exclusion criteria during the search and review phases were resolved by consulting a third author (KGS) to achieve consensus.

### Study quality assessment and certainty of evidence

The evaluation of the methodological quality of the included documents utilized the PEDro scale (44, 45). The PEDro checklist comprises 11 items, with the first item not assigned a rating. Consequently, the scale’s scoring ranges from 0 to 10, with 0 representing the lowest score and 10 the highest. The interpretation of quality assessment was as follows: studies with a score of ≤3 were categorized as having poor quality, those with a score of 4–5 were considered of moderate quality, and those with a score of 6–10 were regarded as high quality. Two assessors (ND and DH) independently assessed the methodological quality of selected papers. Any disparities in their assessments were resolved through discussions and consensus involving a third author (KGS).

The certainty of evidence was analyzed and summarized following the guidelines outlined in the GRADE handbook (46). The same assessors (ND and DH) determined the GRADE level for each selected trial, and any differences in their assessments were recorded and subsequently discussed within our research team until a consensus was reached.

### Data extraction

The data items were common metrics of health-related physical fitness, including (a) body composition (e.g., body mass index (BMI), body fat percentage), (b) muscular strength (e.g., handgrip), (c) muscular endurance (e.g., sit-ups), (d) cardiorespiratory fitness (e.g., six-minute walking test), and (e) flexibility (e.g., sit and reach). Apart from the mentioned data elements, descriptive characteristics of the AVG interventions (e.g., length, frequency) and the participants (e.g., sex, age) were extracted, and adverse effects were recorded.

### Statistical analyses

When at least three trials provided sufficient data to calculate the ES, a meta-analysis was conducted (47, 48). Mean and standard deviation data from before and after the intervention measures were utilized to calculate ESs (i.e., Hedges’ g) for fitness outcomes in both the AVG and control groups. To account for the anticipated heterogeneity among studies, a random-effects model was employed (49). The ES values were presented with 95% confidence intervals (CIs) and interpreted based on the following scale: less than 0.2 was considered trivial, 0.2 to 0.6 was small, 0.6 to 1.2 was moderate, 1.2 to 2.0 was large, 2.0 to 4.0 was very large, and greater than 4.0 was extremely large (50). In trials involving multiple intervention groups, the sample size of the control group was split proportionately so that all subjects could be compared (51). In cases where authors did not submit adequate data (in graphics or were missing), we made efforts to reach out to them through ResearchGate or email the corresponding authors. When data was depicted in a graphical format without accompanying numerical values in tables or Supplementary materials, Graph Digitizer software (DigitizeIt, Germany) was employed to extract the pertinent data from the graphs or figures (52). Heterogeneity across studies was analyzed by using Q and I^2^ statistics. We evaluated the level of statistical heterogeneity using I^2^ statistics. Values below 25% indicated low heterogeneity, 25–75% suggested moderate heterogeneity, and above 75% reflected high heterogeneity (53). Additionally, to assess the robustness of our findings, we performed sensitivity analyses by employing the one-study-removed method. Publication bias was evaluated visually via funnel plots and statistically using Egger’s test (*p* < 0.05) (54).

Subgroup analyses were performed to determine the potential effect of moderator variables on training results. The analyses considered predetermined sources of heterogeneity that could affect the outcomes, namely: training length, training frequency, and session duration. Stratification of the meta-analyses was performed for each of these factors, and a threshold of *p* < 0.05 was utilized as the significance level to determine statistical significance. The Comprehensive Meta-Analysis software (Version 3.0; Biostat, Englewood, NJ, United States) was used for all analyses.

## Results

### Study selection

**Figure 1:**
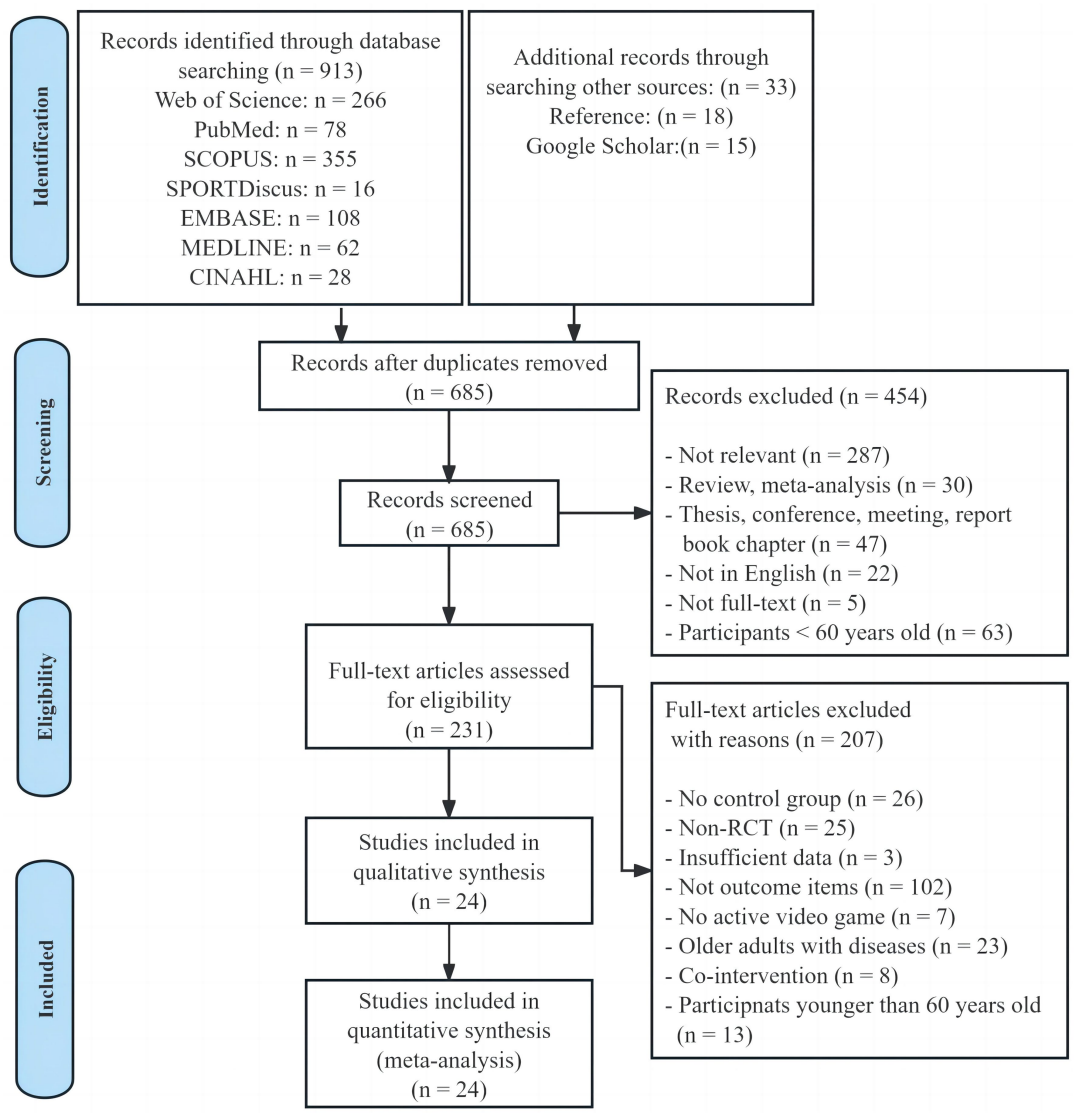
Flow diagram of the study selection.

As depicted in Figure 1, the databases yielded a total of 913 documents, and an additional 33 publications were acquired through references and Google Scholar. After the manual removal of duplicates, there were 685 unique records remaining. The titles and abstracts of these records were assessed, resulting in 231 publications deemed suitable for full-text examination. After a thorough review of all the texts, 207 documents were excluded, leaving 24 trials that fulfilled all the criteria established for the systematic review and meta-analysis.

### Methodological quality and certainty of evidence

According to the PEDro checklist, three studies scored five points, categorizing them as “moderate” quality, while 21 studies scored six to 10 points, indicating their classification as “high” methodological quality (Table 1). Results of the GRADE analyses are provided in Table 2. According to the GRADE assessment, the certainty of the evidence in the analyses ranged from low to moderate.

**Table 1:** Physiotherapy evidence database (PEDro) scores of the reviewed studies.

Authors	Q1	Q2	Q3	Q4	Q5	Q6	Q7	Q8	Q9	Q10	Q11	Total*	Study quality
Maillot et al., 2011 (55)	0	1	0	1	0	0	0	1	1	1	1	6	High
Ray et al., 2012 (56)	1	1	0	1	0	0	0	1	0	1	1	5	Moderate
Jorgensen et al., 2013 (57)	1	1	1	1	0	0	1	1	1	1	1	8	High
Gschwind et al., 2015 (58)	1	1	1	1	0	0	1	1	1	1	1	8	High
Park and Yim, 2015 (59)	1	1	0	1	0	0	0	1	1	1	1	6	High
Sato et al., 2015 (60)	1	1	1	1	0	0	0	1	1	0	1	6	High
Eggenberger et al., 2015 (61)	1	1	1	1	0	0	0	0	1	1	1	6	High
Nagano et al., 2016 (62)	1	1	1	1	0	0	0	1	1	1	1	7	High
Kwok and Pua, 2016 (63)	1	1	1	1	0	0	1	1	1	1	1	8	High
Bacha et al., 2017 (64)	1	1	1	1	0	0	1	1	1	1	1	8	High
Lee et al., 2017 (65)	1	1	0	1	0	0	1	1	1	1	1	7	High
Morat et al., 2019 (66)	1	1	0	0	0	0	0	1	1	1	1	5	Moderate
Yu et al., 2020 (67)	1	1	0	1	0	0	0	1	1	1	1	6	High
Rica et al., 2020 (68)	1	1	1	1	0	0	0	1	0	1	1	6	High
Adcock et al., 2020 (69)	1	1	1	1	0	0	0	1	1	1	1	7	High
Barsasella et al., 2021 (70)	1	1	0	1	0	0	0	1	1	1	1	6	High
Biesek et al., 2021 (71)	1	1	1	1	0	0	0	0	1	1	1	6	High
Sadeghi et al., 2021 (72)	1	1	0	1	0	0	1	1	1	1	1	7	High
Zhao et al., 2022 (73)	1	1	0	1	0	0	0	0	1	1	1	5	Moderate
Gallardo-Meza et al., 2022 (74)	1	1	1	1	0	0	0	0	1	1	1	6	High
Hou and Li, 2022 (75)	1	1	1	1	1	0	0	0	1	1	1	7	High
Lee, 2023 (76)	1	1	0	1	0	0	1	1	1	1	1	7	High
Guede-Rojas et al., 2023 (77)	1	1	1	1	0	0	1	0	1	1	1	7	High
Wang et al., 2023 (78)	1	1	0	0	0	0	1	1	1	1	1	6	High

A detailed explanation for each PEDro scale item can be accessed at https://www.pedro.org.au/english/downloads/pedro-scale. *From a possible maximal punctuation of 10.

**Table 2:** GRADE analyses.

Outcomes	Certainty assessment					No of participants and studies	Certainty of evidence (GRADE)
	Risk of bias	Inconsistency	Indirectness	Imprecision	Risk of publication bias		
Body composition (BMI) follow-up: range 10 to 15 weeks	Serious^a^	Not serious	Not serious	Serious^c^	Not serious	175 (4 studies)	⨁⨁◯◯LOW
Body composition (body fat percentage) follow-up: range 10 to 12 weeks	Serious^a^	Not serious	Not serious	Serious^c^	Not serious	158 (4 studies)	⨁⨁◯◯LOW
Upper body muscular strength follow-up: range 6 to 15 weeks	Not serious	Serious^b^	Not serious	Serious^c^	Not serious	517 (10 studies)	⨁⨁◯◯LOW
Lower body muscular strength follow-up: range 4 to 16 weeks	Not serious	Serious^b^	Not serious	Not serious	Not serious	1,080 (20 studies)	⨁⨁⨁◯MODERATE
Cardiorespiratory fitness follow-up: range 7 to 26 weeks	Not serious	Serious^b^	Not serious	Serious^c^	Not serious	627 (12 studies)	⨁⨁◯◯LOW
Flexibility follow-up: range 6 to 15 weeks	Not serious	Not serious	Not serious	Serious^c^	Not serious	459 (9 studies)	⨁⨁⨁◯MODERATE

GRADE, Grading of Recommendations Assessment, Development and Evaluation; MBI, body mass index. GRADE Working Group grades of evidence High certainty: we are very confident that the true effect lies close to that of the estimate of the effect. Moderate certainty: we are moderately confident in the effect estimate: the true effect is likely to be close to the estimate of the effect, but there is a possibility that it is substantially different. Low certainty: our confidence in the effect estimate is limited: the true effect may be substantially different from the estimate of the effect. Very low certainty: we have very little confidence in the effect estimate, the true effect is likely to be substantially different from the estimate of effect.

a Downgraded by one level due to average PEDro score being moderate (< 6).

b Downgraded by one level due to high impact of statistical heterogeneity (> 75%).

c Downgraded by one level, as < 800 participants were available for a comparison or there was an unclear direction of the effects. Downgraded by two levels in case of imprecision based on both assessed points.

### Study characteristics

Table 3 provides a detailed overview of the participants’ characteristics and AVG programs employed in the included studies. Supplementary Appendix B contains the data used in the meta-analyses. Publications were released between 2011 and 2023. The included studies involved a collective participation of 1,428 subjects, comprising 1,028 females (72%) and 400 males (28%). The sample sizes within the study groups ranged from 15 to 50 subjects, with participants’ ages ranging from 60 to 94 years. Four studies provided information on the effects of AVGs on body composition (56, 67, 68, 73), 22 on muscular strength (55–60, 62, 63, 65–78), 12 on cardiorespiratory fitness (55, 56, 61, 62, 64, 67–70, 75, 77, 78), and nine on flexibility (55, 56, 67, 68, 70, 73, 75, 77, 78). The most commonly used devices in AVG interventions were gaming consoles, such as Nintendo Wii, Xbox 360 with Kinect, and dance mats. The length of AVG interventions ranged from four to 26 weeks. The frequency of AVG sessions ranged from 2 to 3 days per week, and sessions typically lasted between 15 and 90 min.

**Table 3:** Characteristics of the studies examined in the present review.

Author (year)	Participant characteristics			Intervention characteristics				Outcome measures
	n	Age (years)	EG	CG	TL (weeks)	TF (sessions)	SD (min)	
Maillot et al., 2011 (55)	*n* = 32 female (27) male (5)	73.47 ± 3.00 73.47 ± 4.10	Nintendo Wii Fit	No intervention	14	2	60	Muscular strength (arm curls, chair stands), cardiorespiratory fitness (6MWT), flexibility (SAR)
Ray et al., 2012 (56)	*n* = 87 female (58) male (29)	Mean age = 75	Nintendo Wii Fit	No intervention	15	3	45	Body composition (BMI), muscular strength (handgrip, chair stands), cardiorespiratory fitness (6MWT), flexibility (SAR)
Jorgensen et al., 2013 (57)	*n* = 58 female (40) male (18)	75 ± 6	Nintendo Wii	Daily ethylene vinyl acetate insoles	10	2	35 ± 5	Muscular strength (30s chair stand test)
Gschwind et al., 2015 (58)	*n* = 124 female (82) male (42)	EG1: 80.1 ± 6.3 EG2: 82.5 ± 7.0 CG: 80.2 ± 6.5	EG1: Step-mat-training EG2: Kinect games	Educational booklet+usual activities	16	3	20–40	Muscular strength (knee extension)
Park and Yim, 2015 (59)	*n* = 72 female (68) male (4)	EG:72.97 ± 2.98 CG:74.11 ± 2.88	3D VR kayak	Conventional exercise	6	2	20	Muscular strength (handgrip)
Sato et al., 2015 (60)	*n* = 54 female (43) male (11)	69.25 ± 5.41	Kinect games	Normal daily activities	10	2–3	40–60	Muscle strength (30s chair stand test)
Eggenberger et al., 2015 (61)	*n* = 49 female (30) male (19)	77.3 ± 6.3 80.8 ± 4.7	VR video game dancing	Treadmill walking	26	2	60	Cardiorespiratory fitness(6MWT),
Kwok and Pua, 2016 (62)	*n* = 80 female (68) male (12)	70.5 ± 6.7 69.8 ± 7.5	Nintendo Wii Fit	Standard gym- based exercise	12	1	60	Muscular strength (knee extension), cardiorespiratory fitness (6MWT)
Nagano et al., 2016 (63)	*n* = 39 female (12) male (27)	71 ± 5	Step exergames	Daily activities	12	2	15	Muscular strength (quadriceps muscles)
Bacha et al., 2017 (64)	*n* = 46 female (34) male (12)	60–80	Kinect adventures games	Conventional exercises	7	2	60	Cardiorespiratory fitness (6MST)
Lee et al., 2017 (65)	*n* = 40 female (23) male (17)	EG:76.15 ± 4.55 CG:75.71 ± 4.91	Nintendo Wii Fit games	Fall prevention education	6	2	60	Muscular strength (five times sit to stand test)
Morat et al., 2019 (66)	*n* = 45 female (28) male (17)	69.4 ± 5.6	EG1: Dividat Senso stepping exergames (stable) EG2: Dividat Senso stepping exergames (unstable)	Usual activities	8	3	50–52	Muscular strength (leg extension)
Yu et al., 2020 (67)	*n* = 40 female (32) male (8)	64.00 ± 4.44	Kinect adventures games	Normal daily activities	10	3	50	Body composition (BMI, body fat %), muscular strength (handgrip, 30s sit to stand test), flexibility (SAR), cardiorespiratory fitness (6MWT)
Rica et al., 2020 (68)	*n* = 50 female	>60	Kinect games	Board games and normal daily activities	12	3	60	Body composition (BMI, body fat%, lean mass), muscular strength (arm flexion, 30s sit and stand test), cardiorespiratory fitness (800 m walk test), flexibility (SAR),
Adcock et al., 2020 (69)	*n* = 31 female (16) male (15)	EG:77.0 ± 6.4 CG:70.9 ± 5.0	Active home exergame	No intervention	16	3	30–40	Muscular strength (30s sit to stand test), cardiorespiratory fitness (2MST)
Barsasella et al., 2021 (70)	*n* = 60 female (46) male (14)	60–94	VR games	no intervention	6	2	15	Muscular strength (arm curls, 30s sit to stand test), flexibility (SAR), cardiorespiratory fitness (2MST)
Biesek et al., 2021 (71)	*n* = 90 female	71.2 ± 4.5	Nintendo Wii Fit Plus	Usual activities	12	2	50	Body composition (body fat%), muscular strength (handgrip, ankle plantar flexion)
Sadeghi et al., 2021 (72)	*n* = 64 male	71.8 ± 6.09	Sport Xbox Kinect game, your shape fitness game, target kick	Daily activities	8	3	40	Muscular strength (quadriceps muscles)
Zhao et al., 2022 (73)	*n* = 38 female (24) male (14)	65.68 ± 3.78	Nintendo games	Usual activities	12	3	50–55	Body composition (BMI, body fat%), muscular strength (handgrip), flexibility (SAR)
Gallardo-Meza et al., 2022 (74)	*n* = 72 female	EG:69.2 ± 3.7 CG:68.1 ± 3.3	Nintendo Wii	Habitual physical activity	4	2	60	Muscle strength (five-times sit to stand test)
Hou and Li, 2022 (75)	*n* = 44 female (33) male (11)	EG:67.04 ± 3.78 CG:67.52 ± 4.94	DDR game Stepmania	Original lifestyle	12	3	30	Muscular strength (30s chair stand test), cardiorespiratory fitness (6MWT), flexibility (SAR),
Lee, 2023 (76)	*n* = 57 female (26) male (31)	EG:80.39 ± 2.57 CG:79.10 ± 3.90	Nintendo game and Ring Fit Adventure	No intervention	8	3	50	Muscular strength (five-times sit to stand test)
Guede-Rojas et al., 2023 (77)	*n* = 50 female (40) male (10)	EG:71.8 ± 6.8 CG:71.3 ± 7.4	Kinect games	Conventional physical therapy	8	2	60–90	Muscular strength (30s arm curl, 30s chair stand test), flexibility (SAR), cardiorespiratory fitness (2MST),
Wang et al., 2023 (78)	*n* = 98 female (78) male (20)	72.16 ± 4.9	VR games	No intervention	12	2	75–90	Muscular strength (30s arm curl test, 30s chair stand test), flexibility (SAR), cardiorespiratory fitness (2MST)

EG, experimental group; CG, control group; BMI, body mass index; SAR, sit-and-reach; VR, virtual reality; DDR, dance dance revolution; 6MWT, 6-min walk test; 2MST, 2-min stepping test; NR, not reported; TL, training length; TF, training frequency; SD, session duration.

### Meta-analyses

#### The effect of AVGs on body composition

**Figure 2:**
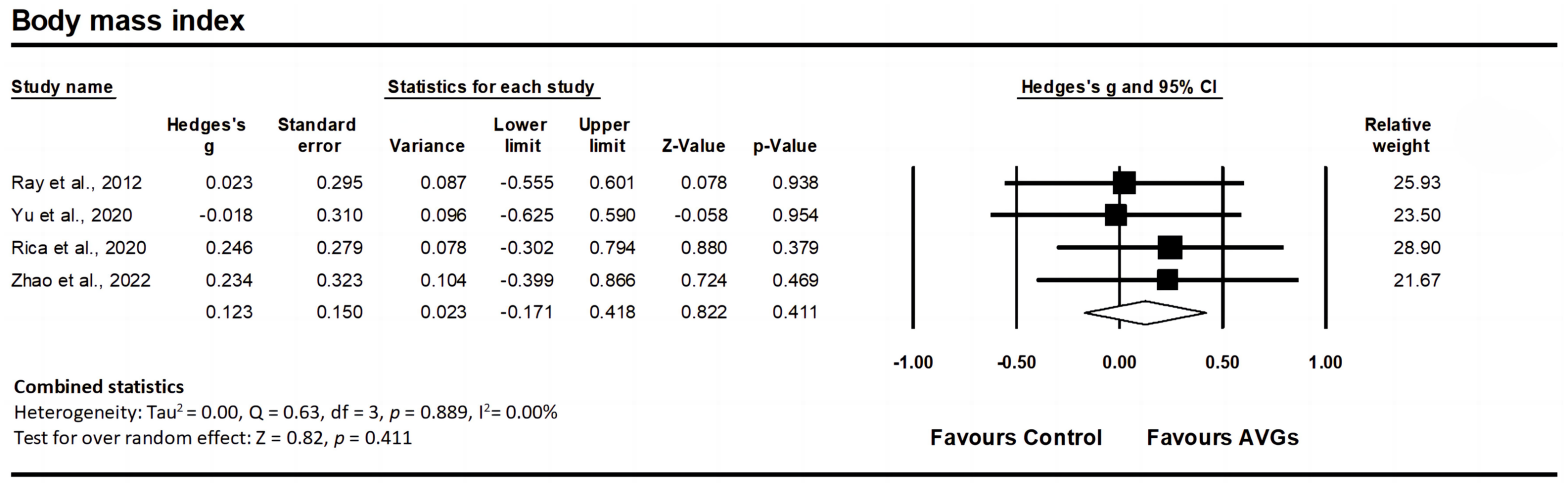
Forest plot of changes in body mass index in older adults participating in active video games (AVGs) compared to controls. Values shown are effect sizes (Hedges’ g) with 95% confidence intervals (CIs). The size of the plotted squares reflects the statistical weight of the study. Effect estimates are based on a random-effects model.

**Figure 3:**
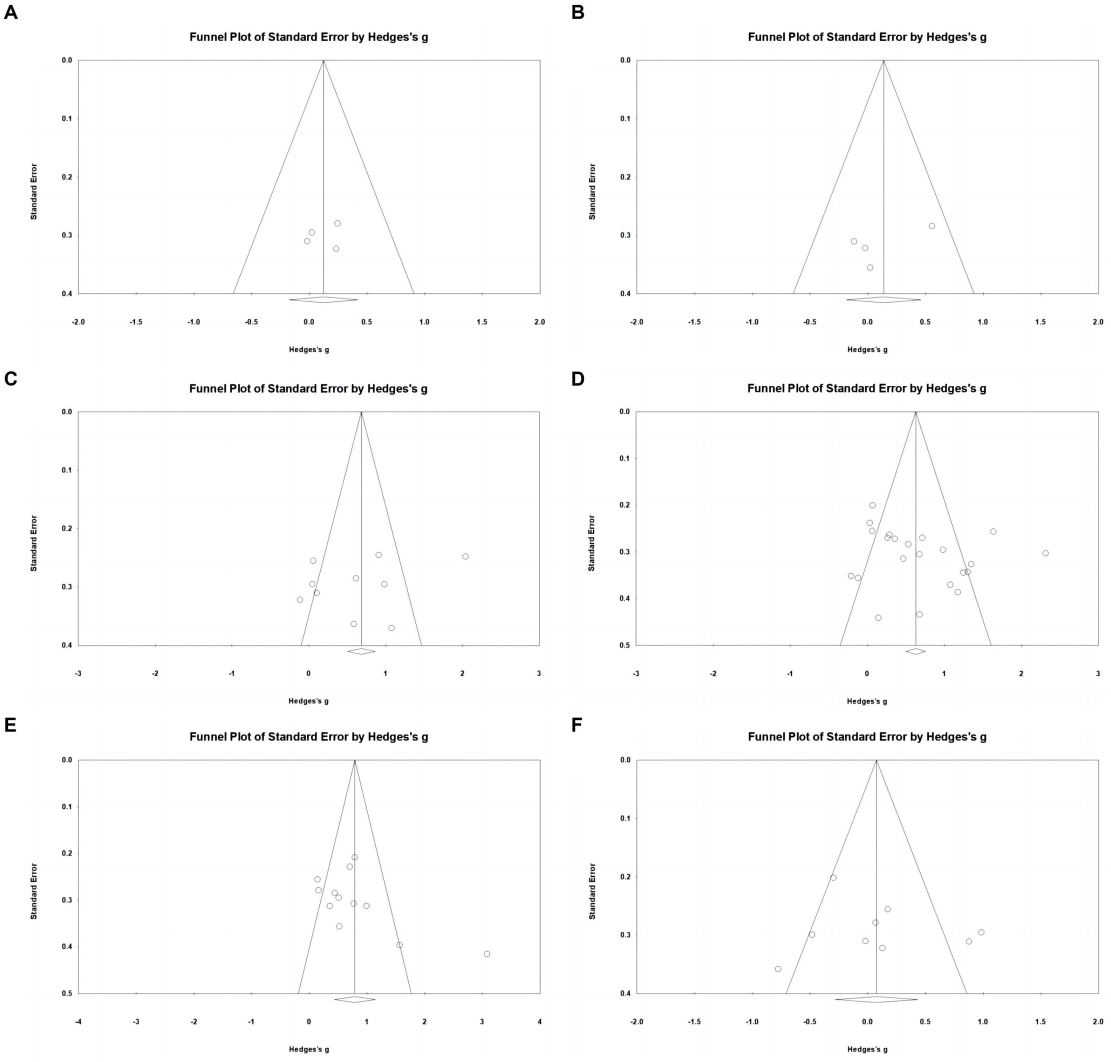
Funnel plots showing risk of publication bias for studies included in the analysis of: **(A)** body mass index; **(B)** body fat percentage; **(C)** upper body muscular strength; **(D)** lower body muscular strength; **(E)** cardiorespiratory fitness; **(F)** flexibility.

Four studies assessed BMI, involving four experimental groups and four control groups (pooled n = 175). There was a non-significant trivial effect of AVGs on BMI (ES = 0.12; 95% CI = −0.17–0.42; *p* = 0.411; Figure 2), with no evidence of heterogeneity between the studies (I^2^ = 0.00%, Q = 0.63, *p* = 0.889). The relative weight of each study in the analysis ranged from 21.67 to 28.90%. The results from the sensitivity analysis were consistent with the original results (Supplementary Appendix C). The inspection of the funnel plot revealed symmetry (Figure 3A), and Egger’s test for MBI did not indicate significant publication bias (*p* = 0.802).

**Figure 4:**
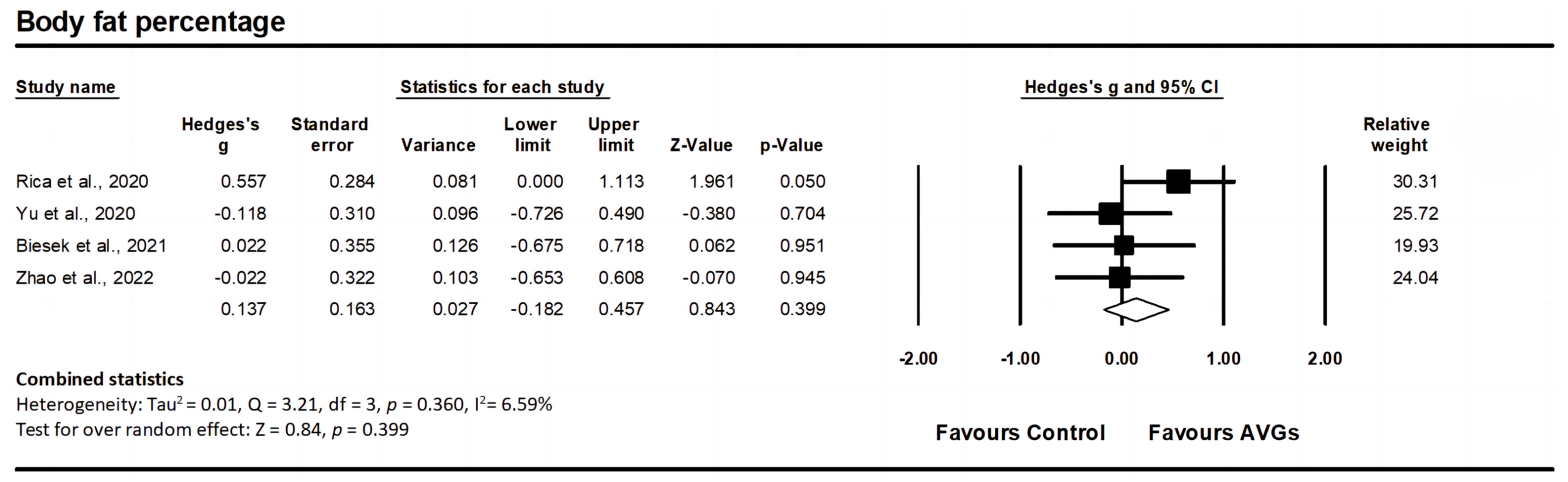
Forest plot of changes in body fat percentage in older adults participating in active video games (AVGs) compared to controls. Values shown are effect sizes (Hedges’ g) with 95% confidence intervals (CIs). The size of the plotted squares reflects the statistical weight of the study. Effect estimates are based on a random-effects model.

Regarding body fat percentage, data from four studies were analyzed, including four experimental groups and four control groups (pooled n = 158). There was a non-significant trivial effect of AVGs on body fat percentage (ES = 0.14; 95% CI = −0.18–0.46; *p* = 0.399; Figure 4), with low heterogeneity between studies (I^2^ = 6.59%, Q = 3.21, *p* = 0.360). The relative weight of each study in the analysis ranged from 19.93 to 30.31%. The results from the sensitivity analysis were consistent with the original results (Supplementary Appendix C). According to the funnel plot (Figure 3B) and Egger’s test result (*p* = 0.314), there was no publication bias for body fat percentage.

#### The effect of AVGs on muscular strength

**Figure 5:**
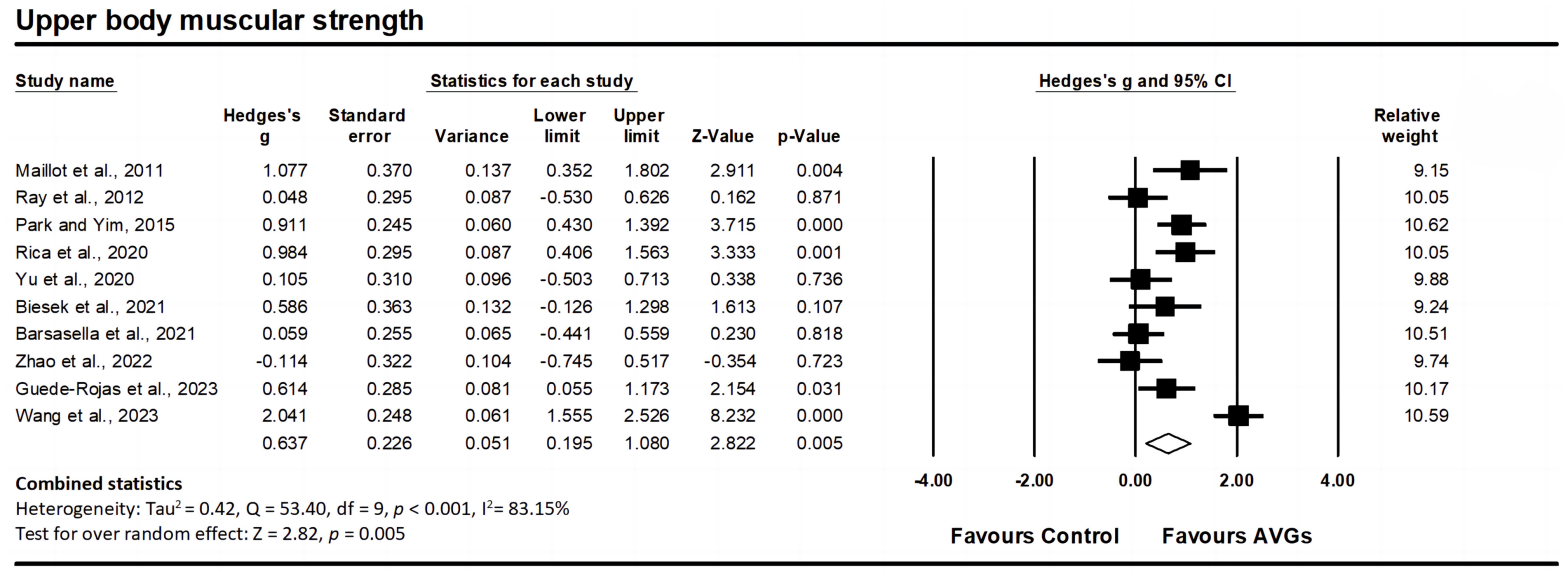
Forest plot of changes in upper body muscular strength in older adults participating in active video games (AVGs) compared to controls. Values shown are effect sizes (Hedges’ g) with 95% confidence intervals (CIs). The size of the plotted squares reflects the statistical weight of the study. Effect estimates are based on a random-effects model.

Ten studies assessed upper body muscular strength, involving 10 experimental groups and 10 control groups (pooled *n* = 517). Results showed a significant moderate effect of AVGs on upper body muscular strength (ES = 0.64; 95% CI = 0.20–1.08; *p* = 0.005; Figure 5), with high heterogeneity between the studies (I^2^ = 83.15%, Q = 53.40, *p* < 0.001). The relative weight of each study in the analysis ranged from 9.15 to 10.62%. The results from the sensitivity analysis were consistent with the original results (Supplementary Appendix C). No significant publication bias was indicated by the funnel plot’s lack of pronounced asymmetry (Figure 3C) and the insignificant result from Egger’s test (*p* = 0.383).

**Figure 6:**
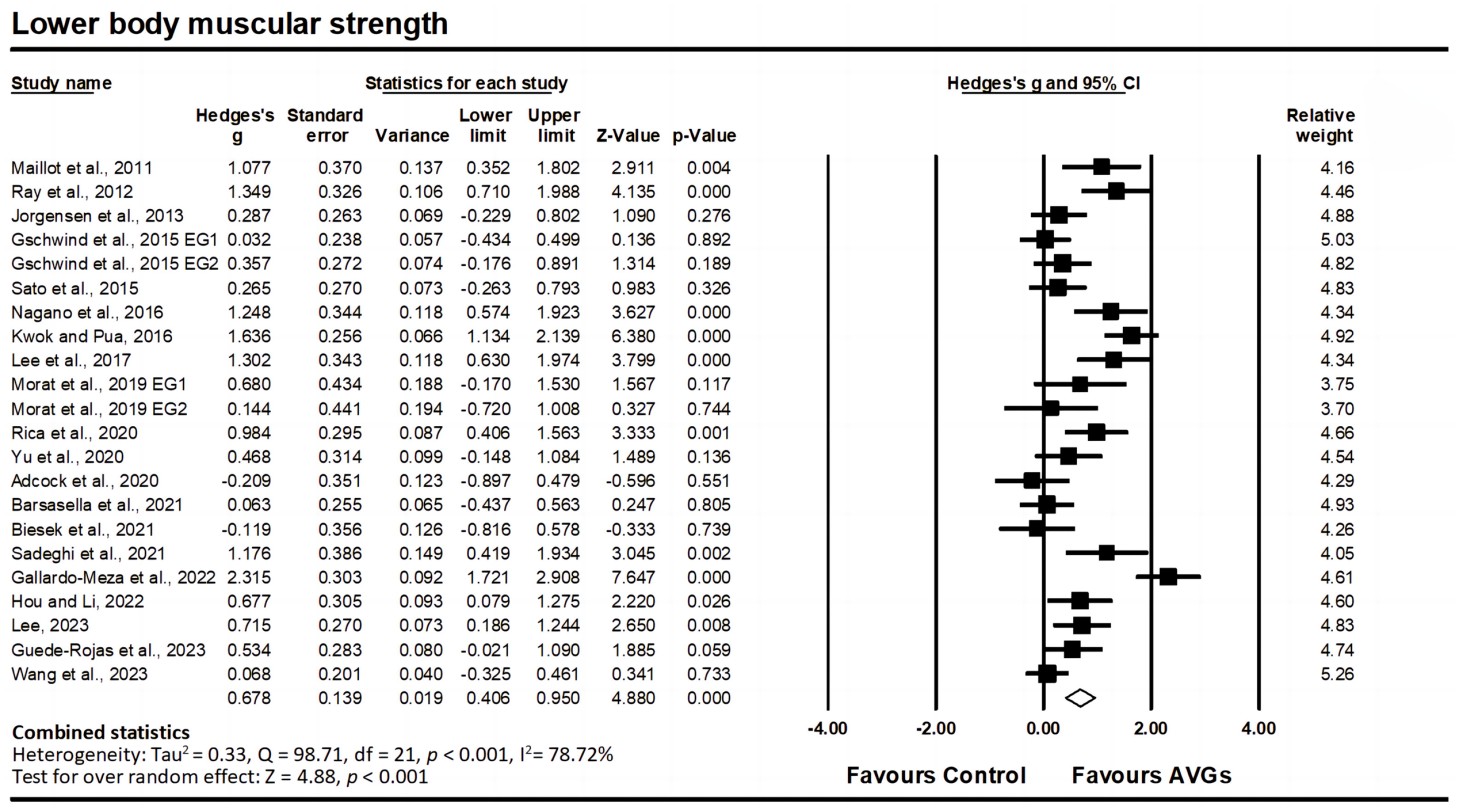
Forest plot of changes in lower body muscular strength in older adults participating in active video games (AVGs) compared to controls. Values shown are effect sizes (Hedges’ g) with 95% confidence intervals (CIs). The size of the plotted squares reflects the statistical weight of the study. Effect estimates are based on a random-effects model. EG, experimental group.

Regarding lower body muscular strength, data from 20 studies were analyzed, including 20 experimental groups and 22 control groups (pooled n = 1,080). Results indicated a significant moderate effect of AVGs on lower body muscular strength (ES = 0.68; 95% CI = 0.41–0.95; *p* < 0.001; Figure 6), with high heterogeneity between the studies (I^2^ = 78.72%, Q = 98.71, *p* < 0.001). The relative weight of each study in the analysis ranged from 3.70 to 5.26%. The results from the sensitivity analysis were consistent with the original results (Supplementary Appendix C). The funnel plot showed no signs of asymmetry (Figure 3D), and Egger’s test yielded a non-significant result (*p* = 0.218), indicating no substantial publication bias.

#### The effect of AVGs on cardiorespiratory fitness

**Figure 7:**
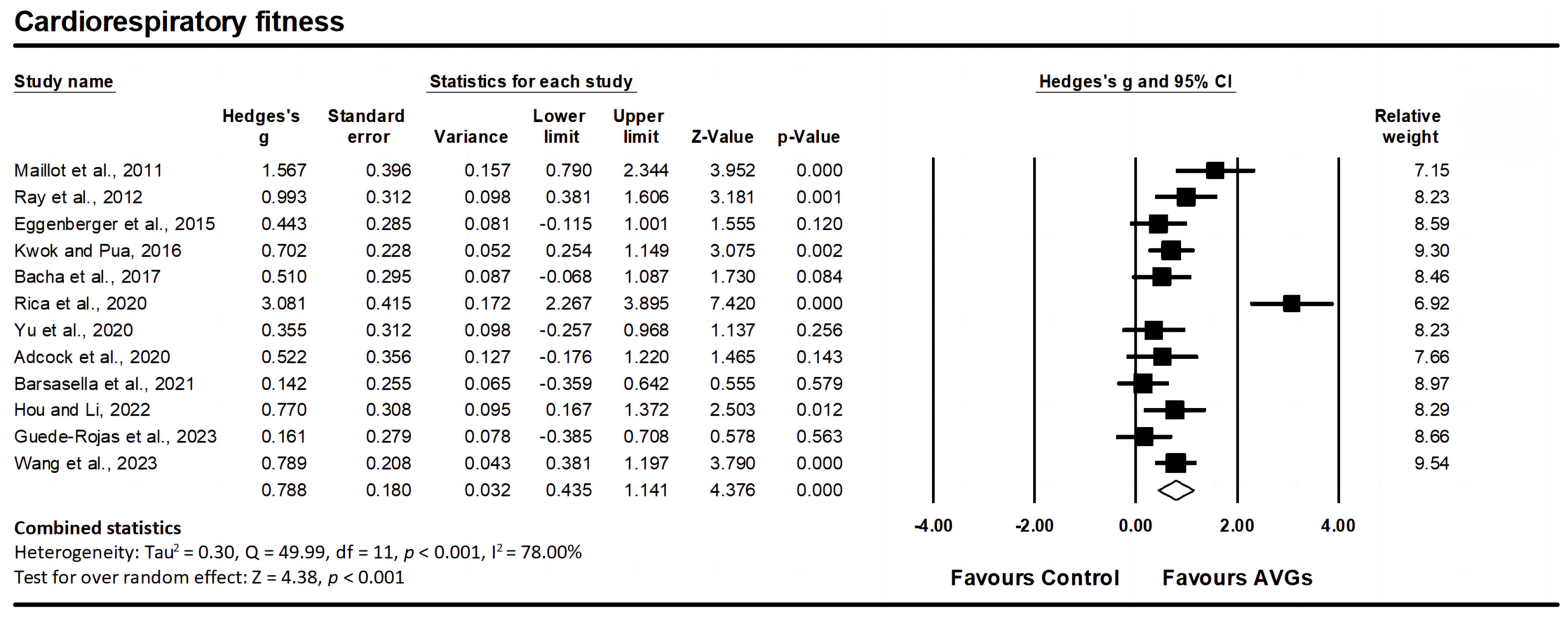
Forest plot of changes in cardiorespiratory fitness in older adults participating in active video games (AVGs) compared to controls. Values shown are effect sizes (Hedges’ g) with 95% confidence intervals (CIs). The size of the plotted squares reflects the statistical weight of the study. Effect estimates are based on a random-effects model.

Twelve studies assessed cardiorespiratory fitness, involving 12 experimental groups and 12 control groups (pooled *n* = 627). Findings indicated a significant moderate effect of AVGs on cardiorespiratory fitness (ES = 0.79; 95% CI = 0.44–1.14; *p* < 0.001; Figure 7), with high heterogeneity between the studies (I^2^ = 78.00%, Q = 49.99, *p* < 0.001). The relative weight of each study in the analysis ranged from 6.92 to 9.54%. The results from the sensitivity analysis were consistent with the original results (Supplementary Appendix C). The funnel plot appeared visually slightly asymmetrical (Figure 3E), suggesting the potential for publication bias. However, this was not confirmed by a non-significant Egger’s intercept value (*p* = 0.088).

#### The effect of AVGs on flexibility

**Figure 8:**
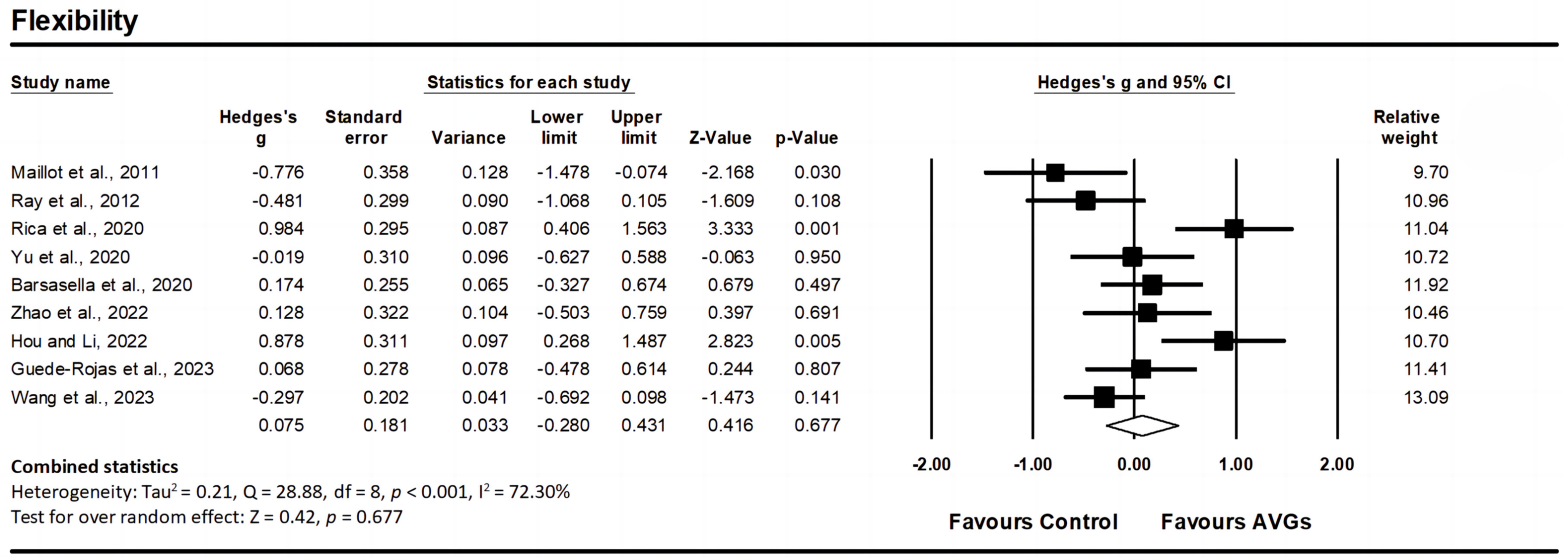
Forest plot of changes in flexibility in older adults participating in active video games (AVGs) compared to controls. Values shown are effect sizes (Hedges’ g) with 95% confidence intervals (CIs). The size of the plotted squares reflects the statistical weight of the study. Effect estimates are based on a random-effects model.

Nine studies assessed flexibility, involving nine experimental groups and nine control groups (pooled *n* = 459). There was a non-significant trivial effect of AVGs on flexibility (ES = 0.08; 95% CI = −0.28–0.43; *p* = 0.677; Figure 8), with high heterogeneity between the studies (I^2^ = 72.30%, Q = 28.88, *p* < 0.001). The relative weight of each study in the analysis ranged from 9.70 to 13.09%. The results from the sensitivity analysis were consistent with the original results (Supplementary Appendix C). The funnel plot displayed symmetry, suggesting an absence of notable publication bias (Figure 3F), and this observation was supported by the non-significant outcome of Egger’s test (*p* = 0.671).

#### Subgroup analyses

A subgroup analysis was performed based on training length (< 12 weeks vs. ≥ 12 weeks), training frequency (2 sessions vs. 3 sessions), and session duration (< 60 min vs. ≥ 60 min) as moderators (Table 4). Regarding training length, a greater improvement in cardiorespiratory fitness (ES = 1.04 vs. 0.29, *p* = 0.028) was noted after ≥12 weeks compared to <12 weeks of AVGs (Table 4). Concerning session duration, a more substantial enhancement in upper body muscular strength (ES = 1.20 vs. 0.27, *p* = 0.007) and lower body muscular strength (ES = 1.24 vs. 0.42, *p* < 0.001) was observed after ≥60 min compared to <60 min session duration of AVGs (Table 4).

**Table 4:** Subgroup analyses of potential moderator factors.

	Groups (n)	ES (95% CI)	I^2^ (%)	p	Q value and (*p*) between groups
Upper body muscular strength					
Training length					
< 12 weeks	4	0.43 (−0.27–1.12)	59.78	0.230	0.60 (0.440)
≥ 12 weeks	6	0.78 (0.20–1.37)	87.65	0.008	
Training frequency					
2 sessions/week	6	0.89 (0.35–1.43)	85.30	0.001	2.08 (0.150)
3 sessions/week	4	0.26 (−0.40–0.92)	63.07	0.442	
Session duration					
< 60 min	6	0.27 (−0.16–0.70)	52.39	0.217	7.17 (**0.007**)
≥ 60 min	4	1.20 (0.67–1.73)	81.44	<0.001	
Lower body muscular strength					
Training length					
< 12 weeks	11	0.65 (0.26–1.04)	81.56	0.001	0.03 (0.859)
≥ 12 weeks	11	0.70 (0.31–1.11)	76.88	<0.001	
Training frequency					
2 sessions/week	9	0.81 (0.40–1.23)	83.71	0.003	1.10 (0.295)
3 sessions/week	11	0.52 (0.36–0.94)	62.61	0.020	
Session duration					
< 60 min	15	0.42 (0.16–0.67)	58.17	0.001	12.28 (**<0.001**)
≥ 60 min	7	1.24 (0.86–1.63)	74.74	<0.001	
Cardiorespiratory fitness					
Training length					
< 12 weeks	4	0.29 (−0.25–0.83)	0.00	0.299	4.81 (**0.028**)
≥ 12 weeks	8	1.04 (0.64–1.43)	80.23	<0.001	
Training frequency					
2 sessions/week	6	0.58 (0.05–1.10)	60.15	0.031	1.62 (0.203)
3 sessions/week	5	1.09 (0.50–1.69)	87.39	<0.001	
Session duration					
< 60 min	5	0.55 (−0.01–1.11)	26.36	0.055	1.20 (0.274)
≥ 60 min	7	0.96 (0.49–1.43)	85.68	<0.001	
Flexibility					
Training length					
< 12 weeks	3	0.08 (−0.58–0.74)	0.00	0.818	0.00 (0.996)
≥ 12 weeks	6	0.08 (−0.40–0.54)	82.51	0.755	
Training frequency					
2 sessions/week	4	−0.18 (−0.68–0.32)	48.25	0.470	1.94 (0.164)
3 sessions/week	5	0.30 (0.16–0.76)	76.46	0.206	
Session duration					
< 60 min	5	0.13 (−0.38–0.65)	60.86	0.608	0.11 (0.738)
≥ 60 min	4	0.00 (−0.56–0.57)	83.35	0.988	

I*^2^*, heterogeneity; *p*, test for overall effect; ES, effect size. The bold values represent statistical significance between subgroups.

### Adverse effects

Ten studies (57, 62–64, 70–74, 77) reported that participants did not experience falls or any possible damages, such as dizziness, nausea, or blurred vision (79), during the training sessions. Of note, two studies reported that participants experienced muscle discomfort, which did not interfere with their normal activities or training schedules. Specifically, Bacha et al. (64) observed that after the first training session, 34% of participants in the AVG group and 26% in the control group reported delayed muscle pain in the lower extremities. Similarly, Gallardo-Meza et al. (74) reported that participants experienced mild muscle soreness after the initial testing and training sessions. However, 14studies included in this review did not mention information on adverse effects (55, 56, 58–61, 65–69, 75, 76, 78).

## Discussion

This meta-analysis investigated peer-reviewed research comparing the impact of AVGs to controls on health-related physical fitness outcomes in older adults. The findings suggest that AVGs were potentially effective in improving muscular strength and cardiorespiratory fitness among older adults. Nevertheless, statistical significance was not reached for the effects of AVGs on body composition and flexibility. Considering the significant heterogeneity observed in most analyses, and the GRADE assessment indicating evidence ranging from low to moderate certainty for the evaluated outcomes, caution is advised when interpreting the results of the meta-analyses.

### The effect of AVGs on body composition

The aging process is linked to alterations in body composition, encompassing both an increase in fat and a decline in muscle mass starting in middle age (80). In this current review, body composition in the studies was primarily evaluated through measures such as BMI and body fat percentage. Our meta-analysis revealed a trivial and statistically insignificant impact of AVGs on body composition in older adults. This finding aligns with results reported by Bourke et al. (81), indicating no observed change in BMI after AVGs in young people compared to the control group. However, several previous meta-analyses focusing on children and adolescents have advocated engaging in physical activity through AVGs as an effective strategy for lowering BMI and reducing body fat (27, 28, 32). Similarly, practicing AVGs has demonstrated the potential to decrease BMI and body fat percentage in individuals who are overweight or obese (82). The variations in study designs, participant demographics, and types of AVGs employed may contribute to the inconsistent results observed in these respective outcomes. Of note, the decline in muscle mass associated with aging (i.e., sarcopenia) has been linked to higher risks of mobility impairment and mortality (83, 84). However, among the studies reviewed, only one explored the impact of AVGs on the lean mass of older adults. The results showed that 12 weeks of AVGs were not effective in promoting an increase in lean mass among older adults (68). In fact, previous studies also did not observe changes in lean mass following an AVG intervention (85, 86). The lack of improvement in body composition observed in our review might be linked to the training protocols, which may have been inadequate in duration (10–15 weeks). Other studies have indicated positive outcomes when older adults engage in physical training for a more extended period (≥ 16 weeks) (87–89). Furthermore, studies have shown that participating in conventional physical activities and implementing dietary control can enhance body composition in older people (90, 91). Additionally, high-intensity training has been reported to effectively reduce visceral and subcutaneous fat (92). Therefore, the potential for AVGs to improve body composition may be realized with long-term implementation or when combined with dietary control and/or higher-intensity game settings. Future studies are encouraged to validate this point.

### The effect of AVGs on muscular strength

Adequate muscular strength is essential for older individuals to manage routine activities such as climbing stairs and carrying groceries (93). The present meta-analysis found that AVGs positively impacted upper body strength in older adults. Similarly, some studies incorporating AVG interventions and fitness tests have demonstrated a significant improvement in upper body muscular strength. For example, an AVG intervention has been shown to positively impact upper body muscular strength in overweight and obese children (94). A virtual dance program effectively enhances upper body muscular strength among community-dwelling older women (95). The enhancement in upper body muscular strength is plausibly ascribed to the intense and frequent arm movements necessitated by playing AVGs. For example, in a virtual reality kayaking game, older adults engage in consecutive and repetitive rowing motions while holding real paddles to navigate the kayak on the screen. This activity may effectively enhance handgrip and upper body strength (59). Playing games on the Nintendo Wii significantly increased upper body strength among older adults (55, 56). This improvement can be attributed to participants being on their feet and utilizing wireless hand-held remotes to play the games (96). However, a meta-analysis by Suleiman-Martos et al. (35) found that the impact of AVGs on grip strength was comparable to that achieved through traditional exercise training. This lack of observed improvement is possibly due to the fact that only a few studies (n = 4) were included for this parameter.

Consistent with earlier systematic reviews and meta-analyses (40, 42, 97, 98), our findings also demonstrate a positive effect of AVGs on lower body muscular strength in older adults. The enhancement in lower body strength is likely attributed to the lower limb movements, and strength demands inherent to AVGs. Engaging in Nintendo Wii games involves participants performing diverse extension and flexion motions of the lower limb joints while supporting their weight on both feet (55–57, 63, 71). The enhanced mechanical function of leg muscles through Wii training is likely a result of training-induced improvements in neuromuscular function, potentially involving an adaptive increase in the size of lower extremity muscles (57). The interactive dance games helped older adults increase muscular strength by practicing movements that involved stepping on the dance mat, maintaining postural control, and changing positions (75). Similarly, in step mat games, participants are required to shift their center of gravity in four directions, potentially leading to improved strength in the quadriceps and gluteus medius muscles (58, 62). Continuous and slow exercises featured in X-box games may also contribute to the improvement of lower limb strength (68). Additionally, the results from the moderator analysis suggest that training sessions lasting ≥60 min are more effective in enhancing muscular strength in older adults. A longer training session may allow more time for sustained physical activity, facilitating a greater stimulus to the muscles (99). Interestingly, Gallou-Guyot and colleagues published an overview of systematic reviews and meta-analyses evaluating the impact of AVGs on the physical functions (e.g., muscular strength) of older adults, revealing highly controversial effects (100). Nonetheless, the findings from our meta-analysis lend support to the potential benefits of AVGs in enhancing muscular strength among older adults.

### The effect of AVGs on cardiorespiratory fitness

Protecting the brain against the normal aging process and the accumulated consequences of age-related health disorders may be possible through enhanced cardiorespiratory fitness (101). We found a moderately positive effect on cardiorespiratory fitness in older adults. The results are in line with previous reviews’ findings (82, 94, 102). The studies incorporated in the present meta-analysis primarily employed AVG protocols (e.g., Nintendo Wii, Kinect adventures, and virtual reality games) lasting 7–26 weeks to investigate their impact on cardiorespiratory fitness (e.g., 6-min walk test). It has been shown that moderate-intensity physical activity can help older people improve their cardiovascular capacity (103, 104). Overall, the intensity of AVGs tended to be light to moderate for older adults; thus, AVGs might influence vascular function and boost cardiorespiratory fitness (98). Furthermore, the underlying biological mechanisms responsible for the heightened cardiorespiratory fitness induced by AVGs may parallel those of conventional physical exercises. These adaptations could involve enhancing the heart’s ability to supply oxygen to active muscles, modifying muscle fibers through mechanical and metabolic stress, promoting protein synthesis, and mitigating apoptosis (98, 105). Additionally, some authors (35) have observed that the effects produced by AVGs on cardiorespiratory fitness do not significantly differ from those obtained with conventional exercise training. However, the robustness of this result is questionable, as the analysis was based on only four studies.

In the existing literature, research on the impact of AVGs on cardiorespiratory fitness is primarily focused on the young population. For instance, a meta-analytic review conducted by Comeras-Chueca et al. (106) revealed notable enhancements in cardiorespiratory fitness among children and adolescents with a healthy weight following AVG engagement. Likewise, another meta-analysis by Comeras-Chueca et al. (82) documented positive outcomes of AVG interventions on cardiorespiratory fitness in children and adolescents with overweight and obesity. Clearly, these positive outcomes align with our findings and support the notion that AVGs could serve as a viable strategy to improve cardiorespiratory fitness. In addition, subgroup analysis for moderator variables indicated that older adults with a longer training duration in playing AVGs were likely to show better improvement in cardiorespiratory fitness. Consistent with our findings, multiple previous studies have illustrated that engaging in AVGs for 12 weeks or more leads to positive impacts on cardiorespiratory fitness across various age groups (107–109). Therefore, it is reasonable to conclude that engaging in AVGs for an extended period (≥ 12 weeks) may be necessary to improve cardiorespiratory fitness in older adults. Given that cardiorespiratory fitness is linked to a reduced risk of all-cause mortality (110), the observed gains in our review hold significant clinical relevance.

### The effect of AVGs on flexibility

Flexibility plays a crucial role in executing both complex and simple movements required for activities of daily living (111). In terms of the effects of AVGs on flexibility, our meta-analysis found that AVGs had a trivial and statistically insignificant impact on older adults’ flexibility. The findings aligned with previous research, which demonstrated that AVGs had no meaningful impact on enhancing flexibility in various cohorts, such as overweight individuals (112) and university students (113). Although sustained whole-body motion is expected to result in increased heart rate and energy expenditure (96), the flexibility of older people did not exhibit a statistically significant improvement. Several aspects of exercise prescription, such as frequency, intensity, and duration, may account for this observation (114). For example, the lack of significant outcomes might be attributed to the short duration of the experimental phase (6–15 weeks) (56, 66, 67, 69, 72, 74). Providing AVG sessions for a longer duration (e.g., more than 15 weeks) may be effective in improving the flexibility of older adults. Moreover, some researchers have explained that participants tended to adopt a more casual approach when engaging with AVGs since these games are designed for relaxation and may not emphasize muscle stretching (67). Additionally, AVGs are not stringent in dictating how older adults play the games, allowing them to exert less effort once they grasp the gameplay (102). Therefore, some authors emphasize that, despite the significant health benefits of playing AVGs, these games may not serve as a substitute for traditional physical activity and real sports (115, 116). Overall, the current available evidence does not support AVGs as an effective means to improve the flexibility of older adults.

### Limitations and future directions

This review presented the latest evidence of the effects of AVGs on the health-related physical fitness of older adults. However, there are some limitations to this review. Firstly, the GRADE assessment indicated that the certainty of evidence for body composition, upper body muscular strength, and cardiorespiratory fitness was classified as low. Secondly, evidence on the effect of AVGs on muscle mass and muscular endurance is limited. Given that muscle mass and muscular endurance are predictive of muscle function and health outcomes in older individuals (117, 118), further research is needed to investigate whether AVGs have positive effects on these factors. Thirdly, older adults have displayed a positive view regarding the physical benefits of AVGs and have expressed a keen interest in adopting AVGs in the future (119). However, the current findings indicate a trivial and statistically insignificant impact on body composition and flexibility. It is recommended that future research focuses on developing AVG interventions that can effectively improve these aspects in older adults. Such efforts could contribute to establishing evidence-based guidelines for this population. Fourthly, substantial heterogeneity was observed in some of the primary and subgroup analyses. Considering the noticeable differences across AVG studies in terms of the subjects recruited, interventions/control conditions employed, and measurement methods adopted, the presence of between-study heterogeneity is not surprising. While the random-effects models used in this review account for statistical heterogeneity, it is essential for future research to identify the specific aspects that may impact the efficacy of AVGs, such as game types, devices, and control group interventions. Finally, 14 trials failed to detail any adverse events linked to AVG interventions, leaving it ambiguous whether there was an effort by the investigators to meticulously document all possible negative responses. Consequently, to broaden our understanding of the safety associated with this training method, it is recommended that future research explicitly disclose any incidents of injuries, discomfort, or other adverse effects stemming from the use of AVGs.

### Practical applications

This review offers insights into optimizing the impact of AVG training courses on the health-related physical fitness of older adults. Practitioners can use AVGs to improve health-related physical fitness, specifically targeting muscular strength and cardiorespiratory fitness in older adults. Subgroup analysis results from our review can help guide the prescription of AVGs for older adults. For cardiorespiratory fitness, the largest effects were observed following long-term AVGs (≥ 12 weeks). The results also indicated that a training session lasting ≥60 min appeared to be more effective in enhancing muscular strength. In addition, training methods based on AVGs are widely accepted and can effectively encourage physical activity among older adults who may lack motivation (35). More well-designed AVG trials involving diverse older adult populations to assess various variables related to fitness outcomes are warranted. This will help identify the optimal design and dosage relationship of AVGs for health-related fitness outcomes.

## Conclusion

This systematic review and meta-analysis provide an overview of the effects of AVGs on health-related physical fitness among older adults. AVGs appear to be an effective tool for enhancing muscular strength and cardiorespiratory fitness in older adults, although their impact on improving body composition and flexibility seems limited. Optimal improvement in cardiorespiratory fitness is associated with a longer duration of AVGs (≥ 12 weeks). Moreover, a session duration of ≥60 min may provide greater benefits for the muscular strength of older adults. More research tailored to older populations is still needed to determine the optimal design and dosage relationship of AVGs for health-related fitness outcomes.

## Data availability statement

The original contributions presented in the study are included in the article/Supplementary material, further inquiries can be directed to the corresponding author.

## Author contributions

ND: Writing – review & editing, Writing – original draft, Visualization, Software, Resources, Methodology, Investigation, Formal analysis, Data curation, Conceptualization. KGS: Writing – review & editing, Visualization, Validation, Supervision, Resources, Methodology, Formal analysis, Conceptualization. BBA: Writing – review & editing, Visualization, Validation, Supervision, Resources, Methodology. HT: Writing – review & editing, Visualization, Validation, Software, Methodology, Formal analysis. DH: Writing – review & editing, Visualization, Validation, Software, Resources, Formal analysis, Data curation.
