# Supplementary material for: Active video games for improving health-related physical fitness in older adults: a systematic review and meta-analysis
Source: Front Public Health. 2024 Apr 17;12:1345244. doi: 10.3389/fpubh.2024.1345244 (PMC11061467; doi:10.3389/fpubh.2024.1345244)
Supplement: Supplementary file 1 [file Table_1.DOCX]

**Table S1. Detailed search strategy.**

**Searched on January 21, 2024**

| **Databases** | **Search strategy** | **Results** |
| --- | --- | --- |
| Web of Science | (AB=(“older adult” OR senior OR elder OR elderly OR “older person” OR “older people” OR gerontological OR geriatric) AND AB=(“virtual reality” OR exergam* OR “video game” OR Wii OR Kinect OR “X-box” OR Nintendo OR PlayStation OR “dance dance revolution” OR “balance board”)) AND AB=(“physical fitness” OR “body composition” OR “body mass” OR BMI OR “body fat” OR “cardiorespiratory fitness” OR “cardiorespiratory endurance” OR “muscular fitness” OR “musculoskeletal fitness” OR “muscle strength” OR “muscular endurance” OR “flexibility”) | 266 |
| PubMed | (("older adult" [Title/Abstract] OR senior [Title/Abstract] OR elder [Title/Abstract] OR elderly [Title/Abstract] OR "older person" [Title/Abstract] OR "older people" [Title/Abstract] OR gerontological [Title/Abstract] OR geriatric [Title/Abstract]) AND ("virtual reality" [Title/Abstract] OR exergam* [Title/Abstract] OR "video game" [Title/Abstract] OR Wii [Title/Abstract] OR Kinect [Title/Abstract] OR "X-box" [Title/Abstract] OR Nintendo [Title/Abstract] OR PlayStation [Title/Abstract] OR "dance dance revolution" [Title/Abstract] OR "balance board" [Title/Abstract])) AND ("physical fitness" [Title/Abstract] OR "body composition" [Title/Abstract] OR "body mass" [Title/Abstract] OR BMI [Title/Abstract] OR "body fat" [Title/Abstract] OR "cardiorespiratory fitness" [Title/Abstract] OR "cardiorespiratory endurance" [Title/Abstract] OR "muscular fitness" [Title/Abstract] OR "musculoskeletal fitness" [Title/Abstract] OR "muscle strength" [Title/Abstract] OR "muscular endurance" [Title/Abstract] OR "flexibility" [Title/Abstract]) | 78 |
| SPORTDiscus | AB ( “older adult” OR senior OR elder OR elderly OR “older person” OR “older people” OR gerontological OR geriatric ) AND AB ( “virtual reality” OR exergam* OR “video game” OR Wii OR Kinect OR “X-box” OR Nintendo OR PlayStation OR “dance dance revolution” OR “balance board” ) AND AB ( “physical fitness” OR “body composition” OR “body mass” OR BMI OR “body fat” OR “cardiorespiratory fitness” OR “cardiorespiratory endurance”OR “muscular fitness” OR “musculoskeletal fitness” OR “muscle strength” OR “muscular endurance” OR “flexibility” ) | 16 |
| SCOPUS | TITLE-ABS-KEY ( "older adult" OR senior OR elder OR elderly OR "older person" OR "older people" OR gerontological OR geriatric ) AND TITLE-ABS-KEY ( "virtual reality" OR exergam* OR "video game" OR Wii OR Kinect OR "X-box" OR Nintendo OR playstation OR "dance dance revolution" OR "balance board" ) AND TITLE-ABS-KEY ( "physical fitness" OR "body composition" OR "body mass" OR BMI OR "body fat" OR "cardiorespiratory fitness" OR "cardiorespiratory endurance" OR "muscular fitness" OR "musculoskeletal fitness" OR "muscle strength" OR "muscular endurance" OR "flexibility" ) | 355 |
| EMBASE | ('older adult':ti,ab,kw OR senior:ti,ab,kw OR elder:ti,ab,kw OR elderly:ti,ab,kw OR 'older person':ti,ab,kw OR 'older people':ti,ab,kw OR gerontological:ti,ab,kw OR geriatric:ti,ab,kw) AND ('virtual reality':ti,ab,kw OR exergam*:ti,ab,kw OR 'video game':ti,ab,kw OR wii:ti,ab,kw OR kinect:ti,ab,kw OR 'x-box':ti,ab,kw OR nintendo:ti,ab,kw OR playstation:ti,ab,kw OR 'dance dance revolution':ti,ab,kw OR 'balance board':ti,ab,kw) AND ('physical fitness':ti,ab,kw OR 'body composition':ti,ab,kw OR 'body mass':ti,ab,kw OR bmi:ti,ab,kw OR 'body fat':ti,ab,kw OR 'cardiorespiratory fitness':ti,ab,kw OR 'cardiorespiratory endurance':ti,ab,kw OR 'muscular fitness':ti,ab,kw OR 'musculoskeletal fitness':ti,ab,kw OR 'muscle strength':ti,ab,kw OR 'muscular endurance':ti,ab,kw OR 'flexibility':ti,ab,kw) | 108 |
| MEDLINE | AB ( “older adult” OR senior OR elder OR elderly OR “older person” OR “older people” OR gerontological OR geriatric ) AND AB ( “virtual reality” OR exergam* OR “video game” OR Wii OR Kinect OR “X-box” OR Nintendo OR PlayStation OR “dance dance revolution” OR “balance board” ) AND AB ( “physical fitness” OR “body composition” OR “body mass” OR BMI OR “body fat” OR “cardiorespiratory fitness” OR “cardiorespiratory endurance”OR “muscular fitness” OR “musculoskeletal fitness” OR “muscle strength” OR “muscular endurance” OR “flexibility” ) | 62 |
| CINAHL | AB ( “older adult” OR senior OR elder OR elderly OR “older person” OR “older people” OR gerontological OR geriatric ) AND AB ( “virtual reality” OR exergam* OR “video game” OR Wii OR Kinect OR “X-box” OR Nintendo OR PlayStation OR “dance dance revolution” OR “balance board” ) AND AB ( “physical fitness” OR “body composition” OR “body mass” OR BMI OR “body fat” OR “cardiorespiratory fitness” OR “cardiorespiratory endurance”OR “muscular fitness” OR “musculoskeletal fitness” OR “muscle strength” OR “muscular endurance” OR “flexibility” ) | 28 |
| Total |  | 913 |
