# Supplementary material for: Active video games for improving health-related physical fitness in older adults: a systematic review and meta-analysis
Source: Front Public Health. 2024 Apr 17;12:1345244. doi: 10.3389/fpubh.2024.1345244 (PMC11061467; doi:10.3389/fpubh.2024.1345244)
Supplement: Supplementary file 2 [file Table_2.DOCX]

| **File 2** | **The data used for meta-analyses** | | |  | | | | |  | | |
| --- | --- | --- | --- | --- | --- | --- | --- | --- | --- | --- | --- |
| **Reference** | **Test** | **EG**  **(pre-test)** | | **EG**  **(post-test)** | |  | **CG**  **(pre-test)** | | **CG**  **(post-test)** | |  |
|  |  | **Mean** | **SD** | **Mean** | **SD** | **n** | **Mean** | **SD** | **Mean** | **SD** | **n** |
| Maillot et al., 2011 | Muscular strength (arm curls) | 16.53 | 3.25 | 19.53 | 2.70 | 16 | 16.8 | 2.57 | 16.8 | 2.73 | 16 |
|  | Muscular strength (chair stands) | 12.93 | 2.66 | 15.66 | 2.28 | 16 | 14.27 | 3.67 | 13.2 | 1.94 | 16 |
|  | Cardiorespiratory fitness (6MWT) | 411.17 | 84.69 | 469.22 | 40.45 | 16 | 429.84 | 61.58 | 432.91 | 26.54 | 16 |
|  | Flexibility (SAR) | 3.67 | 8.17 | 0.89 | 6.20 | 16 | 0.87 | 6.17 | 2.87 | 5.80 | 16 |
| Ray et al., 2012 | Body composition (BMI) | 28.0 | 4.7 | 27.5 | 5.2 | 29 | 29.4 | 1.4 | 29.0 | 1.9 | 18 |
|  | Muscular strength (handgrip) | 23.4 | 8.6 | 24.7 | 7.7 | 29 | 27.3 | 6.1 | 28.2 | 9.0 | 18 |
|  | Muscular strength (chair stands) | 10.0 | 2.3 | 14.4 | 3.0 | 29 | 16.4 | 6.1 | 15.0 | 5.7 | 18 |
|  | Cardiorespiratory fitness (6MWT) | 505.3 | 111.5 | 555.9 | 88.6 | 29 | 578.2 | 121.8 | 448.0 | 268.0 | 18 |
|  | Flexibility (SAR) | -0.4 | 2.5 | -1.8 | 2.8 | 29 | -0.5 | 1.6 | -0.6 | 2.4 | 18 |
| Jorgensen et al., 2013 | Muscular strength (30s chair stand test) | 11.5 | 3.8 | 13.3 | 3.2 | 27 | 11.2 | 3.0 | 12.1 | 3.0 | 30 |
| Gschwind et al., 2015 EG1 | Muscular strength (knee extension) | 24.2 | 10.3 | 25.8 | 9.2 | 39 | 21.9 | 8.6 | 23.8 | 9.1 | 31 |
| Gschwind et al., 2015 EG2 | Muscular strength (knee extension) | 20.8 | 9.4 | 26.2 | 10.3 | 24 | 21.9 | 8.6 | 23.8 | 9.1 | 30 |
| Park and Yim, 2015 | Muscular strength (handgrip) | 21.38 | 5.64 | 23.35 | 4.86 | 36 | 20.39 | 5.52 | 17.73 | 5.19 | 36 |
| Sato et al., 2015 | Muscle strength (30s chair-stand test) | 17.54 | 17.50 | 24.04 | 23.50 | 28 | 19.00 | 18.00 | 19.73 | 19.00 | 26 |
| Eggenberger et al., 2015 | Cardiorespiratory fitness (6MWT) | 505 | 25 | 522 | 21 | 24 | 506 | 18 | 514 | 19 | 25 |
| Nagano et al., 2016 | Muscle strength (quadriceps muscles) | 249 | 74 | 339 | 85 | 20 | 275 | 109 | 276 | 49 | 19 |
| Kwok and Pua, 2016 | Cardiorespiratory fitness (6MWT) | 297.1 | 69.9 | 323.7 | 25.9 | 40 | 290.8 | 85.3 | 335.9 | 26.3 | 40 |
|  | Muscle strength (knee extension) | 24.5 | 8.6 | 30.4 | 2.3 | 40 | 24.9 | 9.4 | 34.6 | 2.3 | 40 |
| Bacha et al., 2017 | Cardiorespiratory fitness (6MWT) | 122.69 | 23.02 | 134.39 | 25.47 | 23 | 121.39 | 24.66 | 144.78 | 19.18 | 23 |
| Lee et al., 2017 | Muscular strength (five times sit to stand test) | 13.63 | 3.87 | 9.31 | 1.81 | 21 | 14.23 | 3.89 | 13.79 | 3.79 | 19 |

| Morat et al., 2019 EG1 | Muscular strength (leg extension) | 4211 | 2200 | 3397 | 1642 | 15 | 2255 | 1306 | 2524 | 1298 | 8 |
| --- | --- | --- | --- | --- | --- | --- | --- | --- | --- | --- | --- |
| Morat et al., 2019 EG1 | Muscular strength (leg extension) | 3825 | 2543 | 4436 | 2590 | 15 | 2255 | 1306 | 2524 | 1298 | 7 |
| Rica et al., 2020 | Body composition (BMI) | 28 | 5 | 27 | 4 | 25 | 28 | 5 | 28 | 4 | 25 |
|  | Body composition (body fat %), | 31 | 5 | 29 | 3 | 25 | 34 | 4 | 34 | 4 | 25 |
|  | Muscular strength (arm flexion) | 21 | 4 | 26 | 4 | 25 | 22 | 4 | 23 | 4 | 25 |
|  | Muscular strength (30s sit to stand test) | 23 | 5 | 29 | 4 | 25 | 24 | 4 | 26 | 4 | 25 |
|  | Flexibility (SAR) | 16 | 2 | 20 | 3 | 25 | 15 | 3 | 16 | 3 | 25 |
|  | cardiopulmonary endurance (800-m walk test) | 17 | 2 | 11 | 1 | 25 | 18 | 3 | 19 | 3 | 25 |
| Yu et al., 2020 | Body composition (BMI) | 23.26 | 2.74 | 23.33 | 2.67 | 20 | 23.27 | 2.79 | 23.29 | 2.78 | 20 |
|  | Body composition (body fat %) | 28.55 | 9.06 | 29.25 | 8.08 | 20 | 32.55 | 5.75 | 32.41 | 5.68 | 20 |
|  | Muscular strength (handgrip) | 27.30 | 8.30 | 27.00 | 10.57 | 20 | 25.85 | 7.20 | 24.60 | 6.82 | 20 |
|  | Muscular strength (30s sit to stand test) | 18.55 | 5.37 | 21.40 | 5.26 | 20 | 16.80 | 4.40 | 17.05 | 5.62 | 20 |
|  | Flexibility (SAR) | 7.75 | 17.60 | 7.35 | 15.38 | 20 | 11.85 | 8.99 | 11.20 | 9.05 | 20 |
|  | Cardiorespiratory fitness (6MWT) | 554.35 | 59.76 | 584.65 | 53.27 | 20 | 534.40 | 72.30 | 539.20 | 84.03 | 20 |
| Adcock et al., 2020 | Cardiorespiratory fitness (2MST) | 66 | 22.5 | 76 | 14 | 15 | 74.5 | 26 | 78.5 | 7.7 | 16 |
|  | Muscular strength (30s sit to stand test) | 13 | 4.5 | 13 | 2.5 | 15 | 16.5 | 6 | 15.5 | 6 | 16 |
| Barsasella et al., 2021 | Muscular strength (30s arm curls) | 28.76 | 9.093 | 32.62 | 9.485 | 29 | 25.45 | 6.324 | 28.68 | 11.548 | 31 |
|  | Muscular strength (30s sit to stand test) | 21.55 | 8.982 | 22.03 | 7.831 | 29 | 19.84 | 7.331 | 19.84 | 7.202 | 31 |
|  | Cardiorespiratory fitness (2MST) | 119.69 | 28.497 | 129.52 | 28.605 | 29 | 109.87 | 24.173 | 116.10 | 21.303 | 31 |
|  | Flexibility (SAR) | 2.369 | 4.020 | 2.091 | 4.620 | 29 | 0.368 | 4.812 | 1.058 | 6.220 | 31 |
| Biesek et al., 2021 | Body composition (body fat%) | 44.9 | 4.1 | 45.1 | 4.3 | 15 | 43.5 | 6.2 | 43.8 | 4.6 | 15 |
|  | Body composition (fat mass) | 32.6 | 7.4 | 32.2 | 6.0 | 15 | 29.6 | 8.3 | 30.2 | 7.3 | 15 |
|  | Muscular strength (handgrip) | 20.1 | 7.2 | 23.3 | 6.2 | 15 | 20.4 | 5.7 | 20.1 | 5.4 | 15 |
|  | Muscular strength (ankle plantar flexion) | 38.2 | 12.8 | 39.6 | 12.1 | 15 | 44.1 | 8.8 | 44.3 | 6.9 | 15 |

| Gallardo-Meza et al., 2022 | Muscular strength (five times sit to stand test) | 0.91 | 0.20 | 1.59 | 0.38 | 35 | 0.89 | 0.31 | 0.84 | 0.23 | 37 |
| --- | --- | --- | --- | --- | --- | --- | --- | --- | --- | --- | --- |
| Sadeghi et al., 2021 | Muscular strength (quadriceps muscles) | 61.9 | 17.0 | 77.2 | 16.9 | 15 | 78.7 | 13.1 | 75.4 | 13.7 | 15 |
| Zhao et al., 2022 | Body composition (BMI) | 23.84 | 2.8 | 23.33 | 2.58 | 22 | 23.65 | 3.69 | 23.86 | 3.54 | 16 |
|  | Body composition (body fat%) | 24.78 | 8.57 | 25.95 | 7.16 | 22 | 25.13 | 6.76 | 26.14 | 6.78 | 16 |
|  | Muscular strength (handgrip) | 28.31 | 8.2 | 27.76 | 9.04 | 22 | 30.30 | 9.1 | 28.70 | 9.0 | 16 |
|  | Flexibility (SAR) | 5.68 | 11.94 | 5.96 | 11.13 | 22 | 5.20 | 11.0 | 6.90 | 10.5 | 16 |
| Hou and Li, 2022 | Muscular strength (30s chair stand test) | 15.17 | 4.38 | 15.58 | 0.98 | 23 | 15.48 | 4.06 | 15.17 | 1.11 | 21 |
|  | Flexibility (SAR) | 5.32 | 7.01 | 5.81 | 0.94 | 23 | 3.04 | 6.90 | 2.69 | 0.94 | 21 |
|  | Cardiorespiratory fitness (6MWT), | 542.89 | 47.95 | 543.52 | 0.88 | 23 | 557.74 | 37.21 | 557.63 | 1.01 | 21 |
| Lee, 2023 | Muscular strength (five times sit to stand test) | 15.87 | 4.33 | 13.09 | 3.83 | 28 | 15.73 | 4.36 | 15.94 | 4.39 | 29 |
| Guede-Rojas et al., 2023 | Muscular strength (30s chair stand test) | 12.6 | 6.2 | 15.1 | 5.0 | 25 | 13.7 | 6.0 | 13.9 | 3.3 | 25 |
|  | Muscular strength (30-s arm curl test) | 10.5 | 3.5 | 14.9 | 3.2 | 25 | 10.4 | 4.0 | 12.4 | 4.4 | 25 |
|  | Flexibility (SAR) | -0.3 | 7.2 | 2.8 | 5.7 | 25 | -1.9 | 7.5 | 0.8 | 5.9 | 25 |
|  | Cardiorespiratory fitness (2MST) | 61.8 | 19.0 | 64.6 | 24.4 | 25 | 64.4 | 14.7 | 63.7 | 17.8 | 25 |
| Wang et al., 2023 | Muscular strength (30s arm curl test) | 18.4 | 3.65 | 24.58 | 4.42 | 48 | 20.54 | 2.94 | 20.1 | 2.77 | 50 |
|  | Muscular strength (30s chair stand test) | 19.42 | 4.12 | 19.75 | 5.04 | 48 | 18.14 | 3.6 | 18.78 | 3.91 | 50 |
|  | Flexibility (SAR) | 4.35 | 9.41 | 7.25 | 9.95 | 48 | 8.43 | 8.18 | 8.75 | 9.02 | 50 |
|  | Cardiorespiratory fitness (2MST), | 99.33 | 10.96 | 110.6 | 14.48 | 48 | 106.74 | 12.74 | 106.7 | 13.96 | 50 |

Note: EG, experimental group; SAR, sit and reach; MBI, body mass index; SD, standard deviation; 6MWT, 2-min walk test; 2MST, 2-min step test.
