# Supplementary material for: Active video games for improving health-related physical fitness in older adults: a systematic review and meta-analysis
Source: Front Public Health. 2024 Apr 17;12:1345244. doi: 10.3389/fpubh.2024.1345244 (PMC11061467; doi:10.3389/fpubh.2024.1345244)
Supplement: Supplementary file 3 [file Table_3.DOCX]

**Appendix C. Results of sensitivity analyses**

**Figure 1.** Results of the sensitivity analysis for body mass index. CI, confidence interval; AVGs, active video games.

**Figure 2.** Results of the sensitivity analysis for body fat percentage. CI, confidence interval; AVGs, active video games.

**Figure 3.** Results of the sensitivity analysis for upper body muscular strength. CI, confidence interval; AVGs, active video games.

**Figure 4.** Results of the sensitivity analysis for upper body muscular strength. CI, confidence interval; AVGs, active video games; EG, experimental group.

**Figure 5.** Results of the sensitivity analysis for cardiorespiratory fitness. CI, confidence interval; AVGs, active video games.

**Figure 6.** Results of the sensitivity analysis for flexibility. CI, confidence interval; AVGs, active video games.
